# Feasibility Assessment of a Fine-Grained Access Control Model on Resource Constrained Sensors

**DOI:** 10.3390/s18020575

**Published:** 2018-02-13

**Authors:** Mikel Uriarte Itzazelaia, Jasone Astorga, Eduardo Jacob, Maider Huarte, Pedro Romaña

**Affiliations:** 1 Nextel S. A., Technological Park of Bizkaia 207B, 1B, 48170 Zamudio, Spain; promana@nextel.es; 2 Department of Communications Engineering, Faculty of Engineering in Bilbao, University of the Basque Country UPV/EHU, Plaza Ingeniero Torres Quevedo, 1, 48013 Bilbao, Spain; jasone.astorga@ehu.es (J.A.); eduardo.jacob@ehu.es (E.J.); maider.huarte@ehu.es (M.H.)

**Keywords:** access control model, fine-grained authorization, constrained device, expressive policy language, least privilege, message exchange protocol, policy codification, sensor, feasibility assessment, performance evaluation

## Abstract

Upcoming smart scenarios enabled by the Internet of Things (IoT) envision smart objects that provide services that can adapt to user behavior or be managed to achieve greater productivity. In such environments, smart things are inexpensive and, therefore, constrained devices. However, they are also critical components because of the importance of the information that they provide. Given this, strong security is a requirement, but not all security mechanisms in general and access control models in particular are feasible. In this paper, we present the feasibility assessment of an access control model that utilizes a hybrid architecture and a policy language that provides dynamic fine-grained policy enforcement in the sensors, which requires an efficient message exchange protocol called Hidra. This experimental performance assessment includes a prototype implementation, a performance evaluation model, the measurements and related discussions, which demonstrate the feasibility and adequacy of the analyzed access control model.

## 1. Introduction

The Internet of Things (IoT) concept embraces an interconnected network of things, the smarter the better, contributing to a higher awareness, enhanced decision making, and more adaptive behavior of systems supporting any business process, integrating pervasive and ubiquitous information and communication technologies. IoT also implies a massive deployment of sensors and actuators, which, with the goal of being inexpensive, are implemented in a range of constrained devices, constrained device sensors (CDSs) herein, classified according to IETF [1], from severely constrained C0 to less constrained C2. Specifically, the IETF defines a range of CDSs from class 0 (C0, less than 10 kB of data and less than 100 kB of code), class 1 (C1, approximately 10 kB data and 100 kB code), to class 2 (C2, 50 kB data and 250 kB code), with different purposes and different features, where Moore’s law [2] is expected to move more slowly and will have a greater impact on price than on capacity [3,4]. Moreover, depending on the use case and location, they may require power autonomy and, therefore, low-power-consumption mechanisms.

In such IoT applications, security needs to tackle the large scale exposure of sensitive information and functionality, and access control, in particular, remains an insufficiently solved problem since current approaches are challenged by divergent properties such as tightness and feasibility. Precisely, traditional security countermeasures cannot be applied directly to CDSs in IoT scenarios because they are too resource consumptive and not optimized for resource-constrained devices. Besides, current feasible E2E access control approaches do not implement an expressive and therefore fine-grained and tight security policy enforcement [5]. In 2016, the Dyn cyberattack [6] caused multiple DDoS attacks to many services originating from devices connected to the Internet such as printers, cameras, etc. This attack is a representative case that demonstrates the insufficiency of the currently implemented security mechanisms in many IP things and the potential impact. Vendors and manufacturers are not paying enough attention to security.

In this paper, we propose the experimental feasibility assessment of an innovative approach proposing an optimized access control model based on an expressive policy language enabling tight enforcement in CDSs, which is fully specified in [7]. This access control model includes a protocol named Hidra that enables secure provisioning and enforcement of dynamic security policies as well as an audit trail, and this protocol is the subject of a performance evaluation driven by a prototype implementation described in this paper.

This paper is an extension of a previous paper entitled “Feasibility assessment of a fine-grained access control model on resource constrained sensors” [8] published in the Spanish Telematic Engineering Conference 2017 (JITEL 2017, 27–29 September 2017, Valencia, Spain). Concretely, in this extension, a full range of policy instances (beyond a unique exemplary one) and derived lengths have been specified to support a more complete impact analysis on the performance of the expressiveness and, therefore, the tightness of the policy to be enforced. The exhaustive policy spectrum used in the trials allows assessing the feasibility of the analyzed access control model in the whole set of envisioned use-cases and scenarios ranging in tightness from the simplest to the most thorough policy enforcement. Additionally, the effectiveness of the proposed policy codification is analyzed. Finally, the details of the Hidra test-bed implementation and the testing model are conveyed, which in the absence of any similar documented experimental analysis, might be used for further empirical performance analysis and benchmarking from now on.

**Figure 1:**
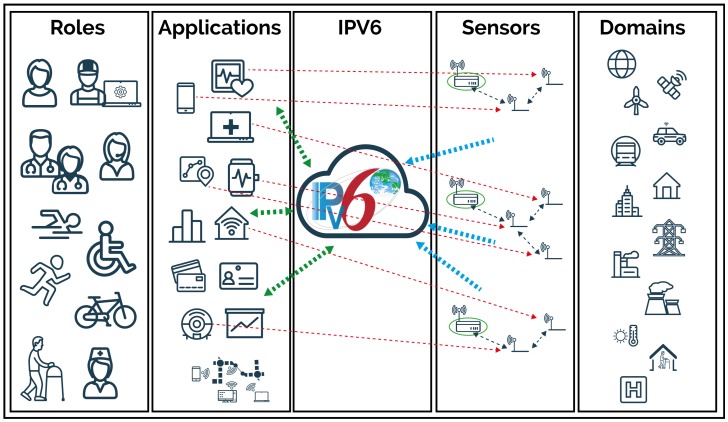
Scenario schema, where several stakeholders playing different roles access E2E IoT applications on different IoT domains through CDSs acting both as simple publishers (thick arrows) and as tiny E2E servers (thin arrows).

Figure 1 shows an IoT schema that conveys different roles in various domains, therein operating, monitoring and controlling related business processes through applications. Beyond the traditional producer behavior of CDSs, which publish measurements and events to message brokers as depicted with thick arrows, in more advanced IoT scenarios, CDSs behave as tiny information servers that can be addressed by their IPv6 address that is natively implemented over Low-Power Wireless Personal Area Networks (6LowPAN) [9]. Specifically, requesting clients directly query the tiny CDS servers, establishing a secure end-to-end (E2E) communication, as depicted with thin arrows. These services that are exposed through the IPv6 network enable the usage, operation, maintenance and manageability of the CDSs over their entire life-cycle and protect the value stream of the connected objects. For example, an end user can utilize direct access to tune personal parameters, such as gender, age, and weight, in a constant health monitoring sensor. In these cases, the use of intermediary proxies is avoided because, on the one hand, they are specific for each protocol or application and are not sufficiently flexible, while, on the other hand, breaking the security association into two or more sub-transmissions might not be considered acceptable from a security point of view.

In this context, the accuracy and correctness of the information exchanged with CDSs are crucial. Protecting this information requires the implementation of appropriate security mechanisms that include fine-grained access control mechanisms based on expressive policies and that can guarantee essential security properties such as confidentiality, integrity, availability, authenticity and non-repudiation [10,11,12]. However, implementing these appropriate security mechanisms in resource-constrained CDSs is not straightforward. Currently, one of the key challenges for enabling the broader adoption of smart things is the availability of feasible access control solutions.

Moreover, due to the extremely dynamic nature and purpose of applications based on services in sensors, policy-based security must be enforced locally in the CDSs, where resources are scarce. That is, besides adherence to the least privilege principle through expressive policies, access control must be able to deal with changes to these policies and application services, and with the fact that it is impossible to know all data, resources and users in advance.

Instead of security rules coupled within the applications’ logic, policy-driven security management has become the de facto approach for security management in large scale systems [13]. In fact, CDSs integrated in IoT are rapidly growing in scale, and at the same time incorporating various emerging technologies. Facing the resulting complexity, traditional system management (including security management) strategies, which mainly rely on IT professionals’ manual work, seem effort-consuming and error-prone for large-scale networks or distributed systems.

To resolve these issues, policy-driven security management is proposed to be leveraged, to simplify the administration of the large scale systems. In a policy-driven management system, administrators just need to specify their targets and constraints in the form of security policies, to guide the behaviours of system elements. From this perspective, a policy can be seen as a common intermediate format, to map requirements of the system to specific and implementable operations.

A core software engineering principle in the study and design of security in general and access control systems in particular is the separation between policy and mechanism, which has its roots in early systems research [14]. The policy is the set of rules that determine what is allowed in the system. The mechanism is the set of software and/or hardware components that know how to enforce the policy in the system.

Specific proposals of access control models for constrained devices from the research community, have addressed authentication in various ways, but authorization has received little consideration. In short, the currently implemented access control mechanisms in CDSs lack sufficient expressiveness of the policy and, therefore, granularity, as well as enforcement features [15,16,17]. There is a proposal of an innovative access control model [7] enabling the enforcement of fine-grained access control policies suitable for C0 and C1 CDSs, but it is mandatory to assess the technical viability and the results are more reliable and realistic if it is done experimentally.

The main contribution of this paper is a feasibility assessment of such a highly expressive E2E access control model in severely constrained devices (C0 and C1 CDSs) based on an experimental performance evaluation. The experimental performance analysis, focusing on three key performance indicators, i.e., the response time, the power consumption and the memory footprint, provides remarkable results. Based on these measurements, the performance evaluation of this proposal demonstrates the feasibility of this analyzed access control model for resource-constrained sensors. Moreover, to the best of our knowledge, no similar experimental performance analysis assessing feasibility has been conducted based on the impact of the tightness of the access control model. Therefore, the proposed test-bed implementation and performance analysis model shall be considered as a reference for future research and benchmarking. Furthermore, on the one hand, the analyzed policy language enables the granting of decisions based on local context conditions defined as rich expressions on attributes. On the other hand, it enables reacting accordingly to the requests by the execution of additional tasks defined as obligations. Consequently, the expressiveness is far beyond the rest of the specifically designed proposals for resource-constrained devices. However, the validation requires to be demonstrated even in the most severely constrained C0 and C1 devices, and this is the purpose of this paper.

The remainder of the paper is organized as follows. Related works are presented in Section 2 as the state of the art. The proposed access control model is specified in Section 3. The performance evaluation model conveying an experimental prototype is described in Section 4, and the resulting performance evaluation is discussed in Section 5. Finally, the main conclusions of the paper are presented in Section 6.

## 2. State of the Art

In the last several years, the research area related to security in IoT has received significant attention, therein addressing the design of different architectures, security protocols and policy models. However, security in general and access control in particular remain the main obstacle in the development of innovative and valuable services [10]. Traditional security countermeasures cannot be applied directly to CDSs in IoT scenarios because they are too resource consumptive and not optimized for resource-constrained devices. Specifically, current feasible E2E access control approaches do not implement an expressive and therefore fine-grained and tight security policy enforcement [5].

For feasibility reasons, a centralized architecture based on traditional standards and protocols, where a central access control server (ACS) with no resource constraints makes authorization decisions for each access request, could initially be a possible option. However, this approach does not consider local context conditions in CDSs, and it implies high energy consumption as well as network overhead due to continuous communications between the CDSs and the ACS.

A recent alternative approach is the distributed capability-based access control (DcapBAC) [18], where an unforgeable token exchangeable as a capability grants access to its holder in a more agile manner. However, the token is designed in an XML schema and has not been validated in resource-constrained devices.

Regardless, this approach has been adopted by other designs involving technologies specifically defined for IoT, which enable CDSs to make local authorization decisions based also on local conditions [19], since the capabilities might include conditions represented as tuples (type, name, value). On the other hand, this approach is based on public key cryptography (PKC), which is more expensive than symmetric key cryptography (SKC) in terms of resource consumption. Additionally, the conditions are limited to simple matching because the approach does not support expressions. Moreover, its syntax is not optimized in terms of codification since it uses JSON; it does not support the enforcement of additional obligations, and it has been validated in not-so-constrained C2 devices.

Along this line, the delegated CoAP [20] authentication and authorization framework (DCAF) [21] defines a token to distribute pre-shared keys, and if authorized, a handshake is performed to establish a DTLS channel. Local authorization policies are specified as conditions serialized in a concise binary object representation (CBOR), instead of JSON, aiming at compacted payloads in the CoAP protocol. However, CBOR is a general-purpose serialization solution [22], and the resulting compression is not sufficiently optimized for security policies in very constrained C0 and C1 devices, where fine-grained access control is achieved through a higher but feasible policy language expressiveness, beyond conditions consisting of the simple constant matching of existing local attributes.

Along the other line, the usage control model and the attribute-based policy schema [23] extend traditional access control systems to a continuous protection of resources during access by the definition of obligations to enforce usage control. However, there are no approaches addressing the feasibility in CDSs.

Finally, concerning protocols specifically optimized for mutual authentication during the E2E security association in a secure session, Ladon [24], which is inspired in Kerberos, has demonstrated its feasibility and security for very-constrained devices C0 and C1 CDSs.

All these aforementioned proposals fail in the expressiveness of the policy because of the infeasibility in the C0 and C1 CDSs. In fact, the challenge of any expressive policy language is to overcome the resource constraints of the CDSs when enabling the definition of fine-grained policies to be provisioned and enforced in the CDS to comply with the least privilege principle. Such a policy language definition would enable both the granting of decisions based on local context conditions and reacting accordingly to the requests by the execution of additional tasks defined as obligations. However, the threat to tackle is to be feasible even in the severely constrained C0 and C1 devices.

Considering that the evolution of Ladon to Hidra [7], Hidra additionally supports the instant provisioning of the policy, enables both the local-context-based fine-grained enforcement on the CDSs and the delegated accounting for improved tracking, auditing and fast anomaly detection. The analytical performance evaluation backs its feasibility [7], however, an experimental performance analysis needs to be conducted to more realistically assess its feasibility and scalability, and this is precisely the scope of this document.

Moreover, to the best of our knowledge, no similar experimental performance analysis assessing feasibility has been conducted based on the impact of the tightness of the access control model. Therefore, the proposed test-bed implementation and performance analysis model shall be considered as a reference for future research and benchmarking.

## 3. Access Control Model

In this section, a brief description of the access control model subject to the feasibility assessment is presented to facilitate the test-bed implementation and performance analysis understanding. Further details are conveyed in [7], but the analyzed E2E access control model is based on an efficient policy language and codification, which are specifically defined to gain expressiveness in the authorization policies and to ensure viability in very-constrained devices. In addition to the policy language, the access control model conveys the E2E security association between two mutually authenticated peers through a security protocol named Hidra. This Hidra protocol, based on symmetric key cryptography, relies on a three-party architecture to enable multi-step authorization as well as the instant provisioning of a dynamic security policy in the CDSs and delegated accounting. Consequently, the CDS is enabled to enforce an ad-hoc context-based security policy not only at the security association establishment but also on every derived resource access during such association. Moreover, the active security policy might be updated under any condition detected through accounting during the security association lifetime, which enables the dynamic revocation and cancellation of permissions.

### 3.1. Access Control Scenario

The considered access control scenario consists of a set of severely constrained CDSs that publish some tiny services. These services on CDSs can be accessed by subjects through IP networks. These CDSs communicate at the link layer with a more powerful base station using specific protocols, such as IEEE 802.15.4 [25], which covers short ranges and enables low bit rates. At the network layer, each CDS implements IPv6/6LoWPAN, enabling the establishment of IP communications with any device in the Internet without the need for intermediary proxies.

**Figure 2:**
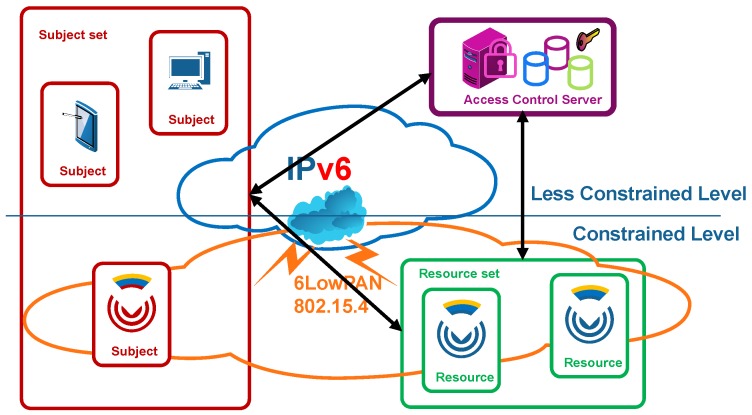
Access control scenario.

There are three basic actors in the access control scenario, as shown in Figure 2: a *subject* (1), constrained or not, which intends to access an either operational or management service as a *resource* in a CDS (2), with the collaboration of a trusted third entity in the establishment of a security association, namely, an *ACS* (3). This schema is aligned with the IoT reference stack [26], where perception layer integrating CDS, and network layers are exploited by the application layer on the top, often through the corresponding graphical user interfaces.

The *subject* endpoint, depicted as a sensor, a smartphone and a workstation, can be a constrained or less constrained endpoint, located in the related application datacenter, or behave as a less trusted mobile endpoint. Note that the term *subject* is adopted as a logical entity, which is a running software or process, acting on behalf of an individual or a service in a machine-to-machine environment. This is a well-known term in access control related models and standards [27,28]. For clarity and uniformity purposes, subject term is used from now on in all aspects of access control model definition, and is equivalent to the client principal term adopted in some standards such as Kerberos [29].

In a similar way, note that the term *resource* is adopted as a logical entity, which is a software component that provides data from or is used in the actuation on physical entities such as constrained devices acting as sensors or actuators. *Resources* typically have correspondent interfaces to interact with them. This is also a well-known term in access control related models and standards. For clarity and uniformity purposes, resource term is used from now on in all aspects of access control model definition, and is equivalent to the service principal and server terms adopted in related standards.

Explicitly, the proposed access control architecture consists of two parts: a standardized cross-domain access control central checkpoint as an initial mandatory filter, namely, the ACS, which is not resource constrained, and a distributed checkpoint in each CDS, which enables dynamic and fine-grained access control, based on local context as well as accounting. This proposed multi-step enforcement approach, which combines centralized and distributed access control architectures, adopts the benefits derived from each and makes E2E security both light and efficient in CDSs.

This ACS, which can be a powerful workstation or a cluster of servers using standard security and traditional access control standards and technologies, supports most resource-intensive features of the Hidra security protocol such as identity and credential management, cross-domain federated authentication, preliminary authorization, policy life cycle management and accounting. Furthermore, the ACS avoids a large number of unsuccessful message exchanges with the CDS when it refuses unauthorized access attempts, and this is a crucial point in terms of energy saving. Additionally, the ACS enables a unified and coherent policy management, which, on the one hand, is becoming a critical aspect in many fully distributed deployments and, on the other hand, reduces storage requirements in the CDS in open and flexible E2E scenarios. Moreover, when a positive preliminary authorization occurs in the ACS, it fetches, wraps, codifies and delivers the proper security policy to be enforced by the CDS based on the local context. The validity of the security policy is related to the security association establishment as well as any further access during its lifetime, and it might convey granting rules related to an individual request, a session, a work-flow etc. in any E2E secure interoperable use case. Moreover, the security policy can be refreshed during the security association lifetime by the ACS under any condition through the Hidra security protocol.

The CDS supports complementary security features such as authentication, security policy reception, secondary local-context-based authorization during security association establishment and lifetime, as well as tracking and accounting notifications. The scope of the feasibility assessment conveyed in this article is the experimental impact analysis on the performance of such features on the CDS that enable E2E dynamic, flexible and tight access control.

### 3.2. Authorization Policy Language and Codification

In this section, a specific expressive policy language is briefly presented. The goal of this policy language is to overcome the resource constraints of the CDSs when enabling the definition of fine-grained policies to be provisioned and enforced in the CDS to comply with the least privilege principle. This policy language definition enables both the granting of decisions based on local context conditions and reacting accordingly to the requests by the execution of additional tasks defined as obligations.

This policy language is declarative and adopts the deontic concepts of rights, prohibitions and obligations. It is inspired by non-constrained access control models, such as XACML, but it balances the expressiveness and feasibility in CDSs adopting and adapting just the core subset of definitions.

A security policy defined with this policy language is an optional set of rules that conveys the conditions to be checked and the derived reactions such as in the broadly adopted event-condition-action approaches. Specifically, this policy language determines a sequence of constructs with particular significance in the decision making and enforcement time. The number and meaning of the constructs in this proposal result from the compromise between expressiveness and performance. The order of the sequence becomes essential to its proper interpretation.

The elasticity of the policy is based on the definition of some constructs as mandatory but some others as optional. This design aspect enables the reduction of the length of the policy when a simple policy is sufficient. In addition, some constructs are extended through other nested constructs, where some of them can be instantiated many times within a container construct. Related to this elasticity feature, the more constructs instantiated, the higher the expressiveness of the policy, the more granular the policy is, and thus the tighter the enforcement is. Consequently, the challenge to overcome is to be feasible even in the most expressive use-case.

Specifically, the proposed policy language enables a security policy instantiation through the *policy* construct, with three nested constructs. First, a policy instance identification *id* is specified for logging, tracking and auditing purposes. Then, a default policy granting *effect* is specified. This effect will prevail in the case of an absence of rules or under any rule evaluation conflict. In the use cases where preliminary authorization enforced by the ACS and mutual authentication is sufficient, this *effect* construct is highly useful, and the resulting policy instance is the simplest and shortest instance. It also becomes very functional for notifying revocation and related security association finalization. Finally, optionally, an array of rules may be instantiated as a *ruleset* to define the conditions and related reactions. Each *rule* in the array is an extensible construct.

**Figure 3:**
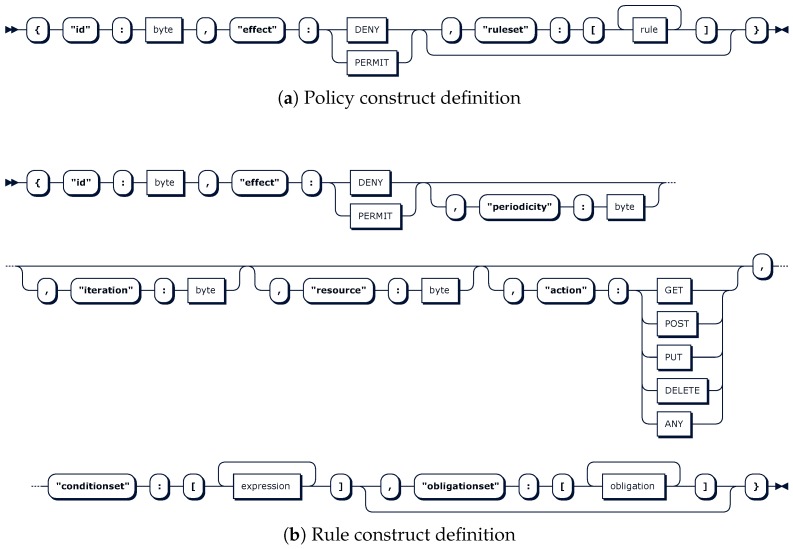
Policy language main constructs.

The representation of the policy construct in the Extended Backus-Naur Form (EBNF) notation is depicted in Table 1. This EBNF representation shows hierarchical constructs to be defined along with expandable, nestable and repetitive constructs as needed. Figure 3a also shows a graphical equivalent view, where optionality and contents are more clearly represented.

The *rule* construct depicted in Figure 3b is defined as a sequence of eight nested constructs, where the order is crucial. Some of them, such as *id*, *effect*, and *conditionset*, are mandatory, and the remainder, namely *periodicity*, *iteration*, *resource*, *action* and *obligationset*, are optional. The *conditionset* and *obligationset* are arrays of expressions and obligations, respectively. These repeatable and extensible *expression* and *obligation* constructs are defined in a similar manner, and they enable the instantiation of rich expressions on attributes declared as inputs as well as reactive tasks declared as obligations. This makes a big difference with currently implemented and researched authorization policy expressiveness and therefore tightness in constrained devices, which convey conditions simply as value matching on a set of attributes. Moreover, there are no constrained-device-oriented approaches supporting obligations, which are enablers of advanced features such as precise and instant logging, active accounting, system and process context updating, system blocking, transaction level control, and usage-based control. In addition, any granting decision on an operational activity can be first triggered and then tracked through rule *id*, *effect*, *resource* and *action* constructs. Finally, optional *periodicity* and *iteration* constructs expand the potential of the obligations.

Consequently, all these constructs nested in the main construct *policy* enable the instantiation of an expressive policy, and it is the purpose of this paper to assess the feasibility according to its performance impact. Moreover, any policy instance is defined in the ACS, provisioned to the CDS when a security association establishment is requested, and enforced before the establishment and during its lifetime in the positive authoritative case.

Likewise, this policy language can be used to define policy instances according to the most broadly implemented access control models (i.e., the RBAC and ABAC models). Any resulting policy instance is a text file that can be generated in several formats, edited by human administrators, and stored and processed by information systems.

The length of any policy instance, in a human-readable format, grows proportionally with the desired tightness, and this would impact the performance negatively. Moreover, the policy expressiveness of the proposed policy language would become useless if the length of the resulting instances was excessive. Therefore, a specific policy instance codification is defined, therein achieving a notable reduction in the length of the policy instances. Considering a policy instance defined according to the proposed policy language, the goal is to generate the minimum possible bit stream. This compression limits the impact of the length of policy instances on both storage and energy consumptions. The proposed codification distinguishes from existing codifications that serialize policy instances through standardized generalist solutions such as CBOR.

The proposed policy codification serializes each construct and concatenates them in a bit stream. The prior knowledge of the defined sequence of the constructs and their format is the cornerstone. An additional and crucial factor is the injection of some agreed-upon bit masks to specify both the existence or not of optional constructs and the number of elements in related arrays of nested constructs. This enables one to deal optimally with the elasticity defined in the policy language, therein avoiding unused but expected fields of expressive policies, which significantly shortens the length. Consequently, the compression ratios are higher than those resulting from currently available generalist approaches such as CBOR.

With respect to covered policy formats, this policy codification can easily be applied to any original textual policy instance format (XACML, JSON, etc.).

Moreover, the agreed-upon conventions can be parametrized for several application scenarios and domains, and the policy domain model (PDM) would be the set of adopted decisions, for example, on the length of the different arrays, the identifiers of functions, and labels. At implementation time, when the PDM is specified in a separate file, its patterning, versioning, modification, provisioning and activation are much more agile, thereby enabling an effective change management. To that end, the better abstraction and multi-domain applicability of the proposed access control model can be obtained, providing scalability and flexibility with the possibility of adjusting the policy expressiveness and scope to the use-case applications and domains.

Regardless, the described policy language and codification are specified in detail in [7].

#### 3.2.1. Resulting Policy Instance Review

When instantiating security policies ranging from simple use cases to highly critical cases, the lengths of the instance files increase progressively due to the elasticity enabled by the policy language. To illustrate such effects, four representative sample groups are conveyed hereinafter.

- Sample 1 (S1): A policy with no rules comparable in expressiveness to traditional approaches broadly implemented and validated in C0 CDSs. This example might be the access granted to a subject initially authenticated and authorized in the ACS and then authenticated in the CDS.
- Sample 2 (S2): A policy containing one rule with conditions, somewhat comparable in expressiveness to the recently researched DcapBAC approaches based on PKC and CBOR policy codifications, which have been validated in less-constrained devices (C2 CDSs). Certainly, the proposed approach under analysis enables rich expressions on attributes such as arithmetic, logical, textual, relational, and comparative, depending on the application domain, rather than only the simple matching of DcapBAC. For example, beyond initial authorization in the ACS and local authentication in the CDS, access for any maintenance action can be granted only after ensuring that the battery level exceeds a given threshold and not matches it.
- Sample 3 (S3): A policy with one rule containing both conditions and obligations. This level of expressiveness is beyond any existing feasible solutions for constrained devices. For example, after checking a local condition, such as the battery status, any maintenance action shall be granted, and, additionally, the system status flag or semaphore is updated as a reaction enabled by obligations.
- Sample 4 (S4): A policy with several rules containing conditions, obligations and periodic re-evaluation. This level of expressiveness is far beyond the capabilities of existing solutions. This example incrementally covers checking different conditions related to different actions on different resources. For example, it can enforce the checking of the system status attribute correlation before maintenance actions can occur and/or the checking of the attempts to perform any operation on the system before granting administration rights. Both conditions highlight its finer granularity. Additionally, each access may produce specific reactions through obligations such as updating system flags to enable transactional controls, updating counters to enable usage controls, or performing concrete remote notifications that enable instant awareness.

To summarize, these four sample groups (S1–S4), defined with a clarifying purpose, convey incremental expressiveness of the policy and therefore granularity and tightness at enforcement time. The four samples range from no rules to several rules to be enforced locally in the CDS, as summarized in Table 2. It is also noted in which constrained platforms each sample range can be used based on currently feasible access control models. The proposed access control model is excluded, and once its feasibility is demonstrated, it would enable all samples in all C0-C2 platforms.

#### 3.2.2. Measurable Policy Instance Examples

For such representative samples, the lengths of specific policy instances vary depending on the specific field values and iterations of the nested constructs that they include. For each of the four samples, exemplary Instances of Samples $\left(IS_{i}\right)$ have been considered to calculate specific lengths.

The policy codification is specified by taking as exemplary input a JSON representation of any policy instance. JSON as an input format is considered appropriate because, on the one hand, it is a broadly adopted format of the payload in CoAP, the de-facto standard web transfer protocol specifically designed for constrained devices [20,30], and, on the other hand, its clarity, being simple and readable by humans.

- Instance sample 1 ($IS_{1}$): An example of a policy instance represented in JSON with no rules but rather a policy identifier and the granting effect for tracking purposes can be instantiated as shown in Listing 1. 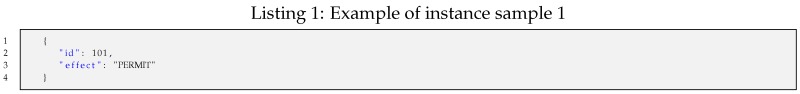  The codification of such a policy instance results in a bit stream of 10 bits packed in two bytes: 0b0110010110.
- Instance sample 2 ($IS_{2}$): An example of a policy instance represented in JSON with one rule enclosing one condition on one input can be instantiated as shown in Listing 2. 
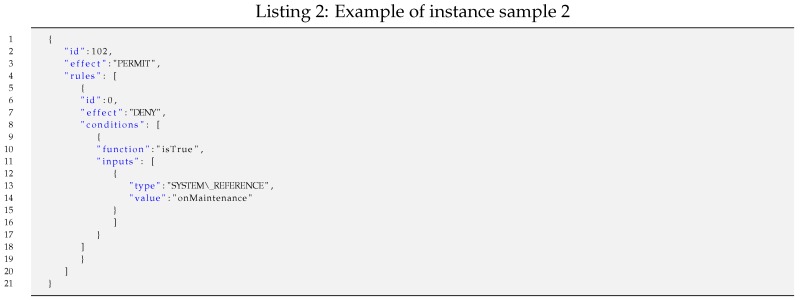
  Such a policy instance shown as a JSON text file is easily readable and manageable for humans. Its length is 164 bytes, but it is shorter in the following minimized JSON format, which is processed by systems.
  {"id":102,"effect":"PERMIT","rules":[{"id":0,"effect":"DENY","conditions":[{"function":"isTrue","inputs":[{"type":"SYSTEM_REFERENCE","value":"onMaintenance"}]}]}]}
  Moreover, such a serialized JSON file can even be further shortened if it is pre-processed by applying some semantic codes, as explained earlier in the policy domain concept. Thus, the length of the resulting optimized JSON file (JSON’) is 118 bytes, which is significantly shorter.
  {"id":102,"effect":"1","rules":[{"id":0,"effect":"0","conditions":[{"function":"160","inputs":[{"type":"6","value":"255"}]}]}]}
  Finally, the codification of such a policy instance results in a bit stream of 53 bits packed in 7 bytes.
- Instance sample 3 ($IS_{3}$): An example of a policy instance represented in JSON with one rule enclosing both one condition on one input and one obligation, which can be instantiated as shown in Listing 3. 
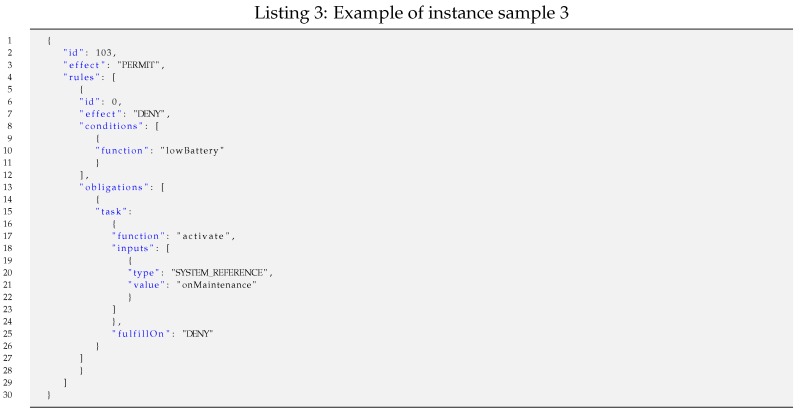
  Such a policy instance shown as a JSON text file (236 bytes) is shorter in a minimized JSON format and can be made even shorter if it is pre-processed by applying some semantic codes, as shown in earlier examples. Thus, the length of the resulting optimized JSON file (JSON’) is 174 bytes, which is significantly shorter.
  The codification of such a policy instance results in a bit stream of 67 bits packed in 9 bytes.
- Instance sample 4 ($IS_{4}$): An example of a policy instance represented in JSON with two rules and periodic re-evaluation. The first rule contains one condition with no input and one obligation. The second rule contains three conditions on up to three inputs and one obligation. Such a policy instance can be instantiated as shown in Listing 4. 
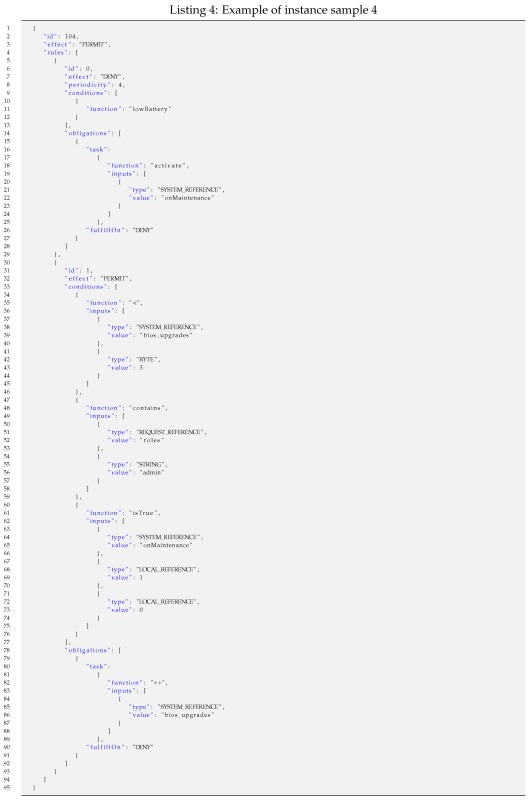
  Such a policy instance shown as a JSON text file (798 bytes) is shorter in a minimized JSON format and can be made even shorter if it is pre-processed by applying some semantic codes, as shown in earlier examples. Thus, the length of the resulting optimized JSON file (JSON’) is 554 bytes, which is significantly shorter.
  The codification of such a policy instance results in a bit stream of 258 bits packed in 32 bytes. This example is illustrative of the incremental effectiveness of the proposed codification.

The length of the policy instances grows significantly with the tightness of the enforcement. Table 3 shows the detailed lengths of the four examples of sample instances previously presented ($IS_{i}$) and their expressiveness in terms of enclosed constructs.

To assess the compression factor of the proposed codification, it is also pertinent to compare the resulting lengths with the existing representations and serializations of the policy instances. The exemplary sample instances ($IS_{i}$) as represented in JSON and CBOR result in the lengths shown in Table 4, where the authorization policy binary representation proposed in the access control model under the feasibility assessment is denoted by the Authorization Policy Binary Representation (APBR).

According to the resulting lengths shown in Table 4, the JSON format policy instances, which are the de-facto standard adopted in CoAP, are excessively long in comparison. Furthermore, after pre-processing the JSON representation with identifiers and PDM-related semantic conventions, the resulting lengths are still excessive. The CBOR format results in shorter lengths than JSON, but they are still longer than the proposed approach because they are not optimized for policy specification and interpretation purposes. Consequently, the proposed policy codification, APBR, achieves the best compression factor. APBR is the shortest; therefore, it can be anticipated to produce the smallest impact on storage, transmission and processing.

### 3.3. Hidra Messaging Protocol

**Figure 4:**
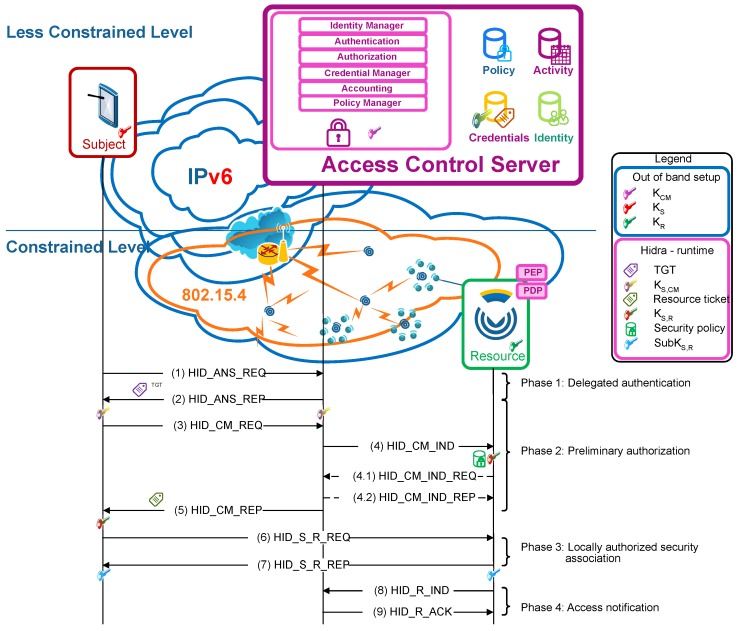
Hidra protocol.

To efficiently convey the presented access control policies to the CDSs, the Hidra protocol is considered. Hidra, depicted in Figure 4, is based on a three-party architecture and provides authentication and authorization in two steps: dynamic policy provisioning and accounting.

Hidra is based on Ladon [24], which is a validated solution for C0 CDSs in the establishment of E2E security associations, through pair-wise keys, guaranteeing mutual authentication and very basic authorization (S1 and S2 samples) but lacking provisioning and accounting features.

Both Hidra and Ladon are based on symmetric key cryptography, and they assume that each endpoint possesses a secret key shared with the ACS. The operation is based on the use of tickets, a capability distributed by the ACS that contains a proof of the identity of the subject that requests it. Tickets are encrypted so that only the entities for which they are intended are able to decrypt them.

After a successful authentication in the ACS (Phase 1), the subject that wants to access a service in the CDS obtains a ticket granting ticket (TGT). This TGT is used by the subject to demonstrate the authentication to obtain resource tickets (Phase 2) required to access any resource on the CDSs.

This approach enables attribute-based access control (ABAC) authorization enforcement in two steps. In the first step, as a condition for releasing any resource ticket, fine-grained preliminary access control is performed in the ACS (Phase 2), focusing on the attributes of the subject, resource and expected actions. If this first authorization step is successful, the ACS sends a message to the subject including a resource ticket; it also sends a message to the CDS, which in addition to the session key with the subject conveys an expressive authorization policy instance. This particular instantaneous custom policy provisioning avoids the permanent storage of policies in the CDS and reduces network overhead compared with approaches enclosing the policy in the resource ticket. Typically, policies are enclosed in the resource ticket. However, such an approach implies increasing the length of the ticket, which is a long structure by itself and can result in packet fragmentation and thus additional network overload due to the short available payloads of IEEE 802.15.4 frames. Therefore, there is an advantage for the HID_CM_IND message, which is one of the smallest messages, to efficiently convey the access control policy to the CDS.

In the second authoritative step, once the subject has obtained a resource ticket, the local context-based access control is performed in the CDS (Phase 3). First, the proper rule is evaluated to make the granting decision, and, then, the corresponding reactive actions are enforced. In a positive authorization case, the security association is established, and the result is a shared session key to be used for future E2E resource access exchanges.

Another remarkable feature of Hidra is the addition of a pair of messages to enable precise accounting (Phase 4). With these messages, the CDS will provide notification of details, such as who performed what, where and when, in every access request received from the subject. These notifications are gathered, normalized, and treated properly by the ACS. Additionally, the ACS can react and send a related policy message, enabling the dynamic delegation, request, cancellation and revocation of permissions.

Then, although the security association is not finalized, the access control is enforced in the CDS autonomously in every future request attempt since the received expressive policy (Phase 2) includes related rules.

Consequently, the unified, coherent and adaptive management of the policies by the ACS is achieved. Additionally, the proposed Hidra protocol and the adopted architecture enable one to relay the most expensive features on the ACS, which entails the usage of standard security and access control technologies in non-constrained interactions. It also ensures that most unauthorized access attempts are refused before reaching the CDS, avoiding unsuccessful message exchanges and thus saving energy in the CDS, which is a crucial aspect.

## 4. Performance Evaluation Model

The performance analysis of the access control model that leads to the final feasibility assessment shall be conducted in three ways: analytical evaluation, generic or specific network simulation and prototype implementation. An analytical performance evaluation, which covers the computation of the crucial theoretical performance parameters referring to a concrete scenario and under a set of assumptions is conveyed in [7].

There are basically four criteria considered for the three remaining methods. The first and most relevant criterion is the veracity of the resulting data. The other three criteria are the agility of the software development, the scalability of the deployment and the reuse of the software. Therefore, a real test-bed will be implemented using sensor nodes and hardware hosts. Even if it requires a larger effort and lacks broad testing scalability, the resulting testing results are more valuable and conclusive for the final feasibility assessment.

### 4.1. Hidra Test-Bed Implementation

The Hidra test-bed implementation is described in the following, which also presents the reference scenario conveying the platform selection as well as the implementation through the software codification and configurations.

#### 4.1.1. Hidra Test-Bed Scenario

**Figure 5:**
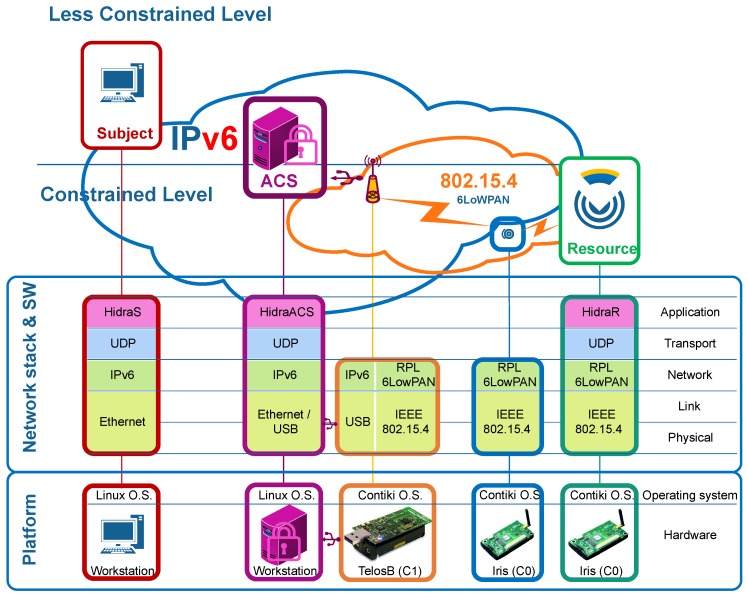
Performance evaluation scenario. Hidra protocol implementation conveying the hardware, operating system, network stack and Hidra software modules.

The test scenario for the experimental performance evaluation is graphically depicted in Figure 5. In this scenario, a subject is connected to the Internet and establishes an E2E connection with a resource running as a tiny server on a CDS in an IEEE 802.15.4 network in the 2.4 GHz band. A 6LoWPAN router (in orange) acts as the LowPAN coordinator and connects a beacon-enabled lineal structure to the Internet. The IEEE 802.15.4 network is 2-hops deep, which is considered significantly large for validation purposes. The PAN router coordinator has a child coordinator, which controls one leaf node. In this node, the CDS exposes resources as management services.

Both the coordinator router and the sensor node are class C0 and C1 CDSs [1], and thus, proper operating systems and hardware platforms shall be selected for this purpose. Neither traditional general-purpose operating systems nor real-time operating systems are suitable for typical applications on CDSs. Therefore, the selected platform focuses on operating systems specifically designed for CDSs, aiming at higher efficiency, flexibility, portability and lightness as well as smaller footprints.

The operating system should be easily programmable and configurable, and it should be based on low complexity operations because of the low cycle speeds of the micro-controllers (MCU) of the CDSs. Therefore, the key features are the kernel architecture (monolithic, layered or multi-layered), the programming model (in terms of parallelism, memory hierarchy and concurrency), the scheduling strategy (real time and multitasking support), networking (light-weight communication protocol support), memory management (static versus dynamic) and portability to different hardware platforms. All these features significantly impact the performance, programmability as well as the manageability; for various use cases, many operating systems are being proposed and implemented in several IoT platforms.

From the research point of view, considering the openness, maturity and the adoption by the community as well as the technical features described in [31], three operating system have been examined: TinyOS, Contiki OS and Riot OS, as summarized in Table 5. The technical features of Riot OS are the most complete and promising, but according to a broad IoT developer survey of 2017 [32], current adoption and research community support are higher for Contiki OS, and therefore, Contiki OS is the selected platform.

Contiki OS [33] is an open-source operating system for sensor nodes. Contiki OS combines the advantages of events and threads, therein implementing a hybrid *Protothread* model, which supports both event-driven and multi-threading operation. Contiki OS comes with a library that offers pre-emptive multi-threading on top of the event-driven kernel. The library is only linked with the program if an application explicitly uses it.

The Contiki OS memory has a footprint of 1.5 kB, and the OS is written in the C language, with the inclusion of its own adaptation directives for the use of threads and the hardware functionalities of the sensors. This OS’s kernel allows direct communication with the hardware by the drivers. There are two partitions in the OS: the core and the loaded program. As a general rule, the core is not changed once loaded, while the loaded program can be modified at run time.

The implementation of the energy management is not performed natively by the OS; it must be implemented through a series of developer-provided functions.

Contiki OS has been ported to a number of mote platforms based on different microcontrollers. For communication, Contiki OS includes microIP (uIP) and Rime stacks. The uIP stack is a light-weight stack that supports IPv6. The uIP stack uses protocols specifically deployed for low-power and wireless memory-constrained real-time sensor devices.

Once the operating system is selected, compatible C0 and C1 hardware platform sensors are analyzed. The considered sensors are TelosB [34], Zolertia Z1 [35], Wismote [36] and IRIS [37]. The selection criteria are the technical features, broad adoption, the programming potential and usability. These four criteria are appropriate for the prototype, and there is no clear winner. Therefore, IRIS is selected for implementing the sensor-side Hidra protocol and TelosB for implementing the border router because of the broad adoption, availability and previous background. Their technical features are summarized in Table 6.

#### 4.1.2. Hidra Security Protocol Application Implementation

In the software implementation, two aspects are distinguished: on the one hand, network protocols and the required E2E connectivity, and, on the other hand, the Hidra messaging security protocol running at the application layer. The Hidra messaging protocol application is achieved by different software modules covering different functions for each of the three parties involved in the protocol: subject, ACS, and sensor.

IPv6 connectivity is depicted in Figure 5, which shows implemented protocol and software stacks in all entities involved in the performance evaluation scenario. The implemented network enables E2E IPv6 connectivity through a multi-hop IEEE 802.15.4 sensor network. It is straightforward in the workstations, and the border router, whereas the border routing sensor, the coordinator node and the endpoint CDS node require the proper installation and configuration of such IPv6 routing. Note that IPv6 Routing Protocol for Low-Power and Lossy Networks (RPL) [38] is configured in the sensor nodes to set a multi-hop hierarchical node network with neighbor discovery functionalities within the IEEE 802.15.4 network.

**Figure 6:**
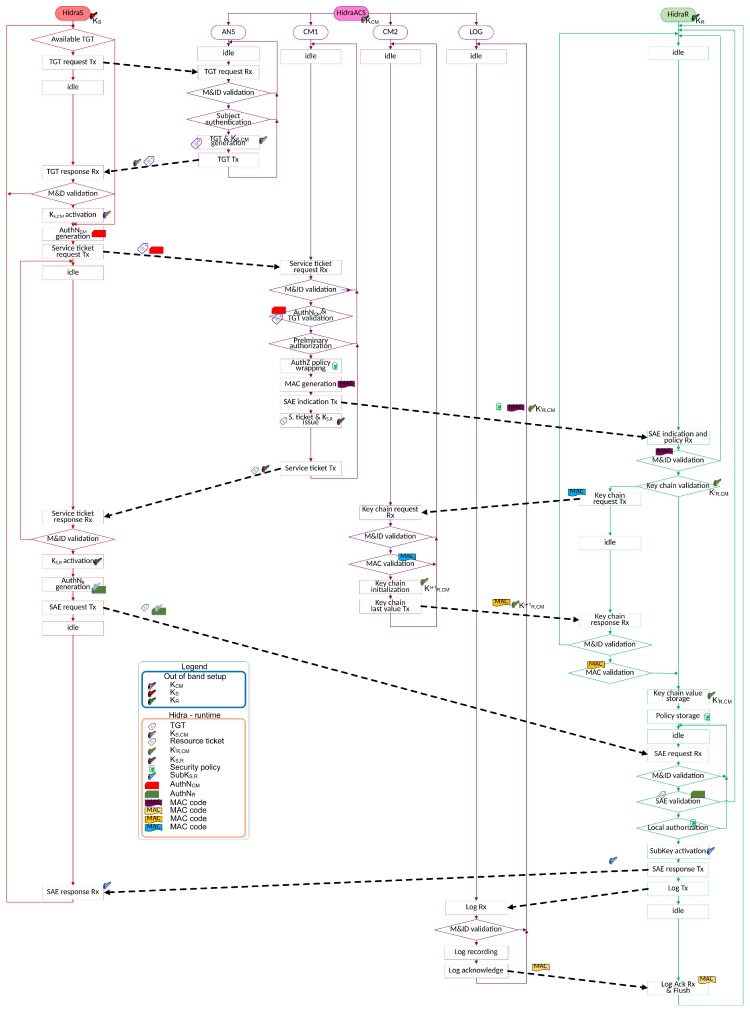
Hidra software flow diagram.

The three different modules of the Hidra software described herein and their interactions are depicted in Figure 6, which notes main entities such as tickets, keys, session authenticator blocks as well as message authentication codes. Certain details, such as lifetimes, nonces, and IDs, are not represented for clarity purposes.

- Hidra in the ACS side (*HidraACS*) is similar to the Key Distribution Center (KDC) in Kerberos, and it is made by different sub-modules listening in particular UDP ports: ANS, CM1, CM2 and LOG. Additionally, four different repositories are distinguished to support the management of identities, credentials, policies, active connections and logs.
  – The ANS module (*HidraACS.ANS*) is the authentication server in the ACS. For this purpose, an UDP socket is open at port 8888. Upon reception of every authentication request message, first, it checks if the message is for the ANS; then, it checks the identity of the subject on the repository, as shown in Listing 5. In the positive case, it generates a long-term ticket granting ticket (TGT) ciphered with the CM’s secret key $K_{CM}$. It also cyphers with the subject’s secret key $K_{S}$ a data block containing the shared key $K_{S,CM}$ and a set of nonces for further sequence validation. It also records such a TGT in the active connection repository with a $Nonce_{S,CM}$ and the corresponding lifetime. 
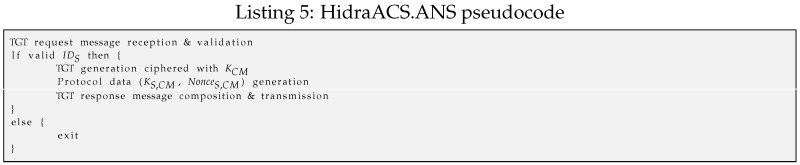
  – The CM1 (*HidraACS.CM1*) module is the credential manager. For this purpose, a UDP socket is open at port 8899. When this sub-module receives a subject’s request to access a resource on the CDS, first, it checks the validity of the addressed CDS, as shown in Listing 6. Then, it deciphers the TGT and checks the validity of the request checking the $Nonce_{S,CM}$. Then, it enforces the preliminary authorization supported by the authorization server AZS. In the positive case, it fetches and codifies the proper policy instance to be sent to the CDS in an indicative message HID_CM_IND. In this indicative message, to assure its freshness, a new key value $K_{S,CM}^{i}$ from a previously existing or just initiated one-way key chain [$K_{S,CM}^{1}\ldots K_{S,CM}^{N}$] is enclosed. It also responds to the subject with another message enclosing an encrypted service ticket only readable by the CDS. It finally records such a service ticket in the active session repository.
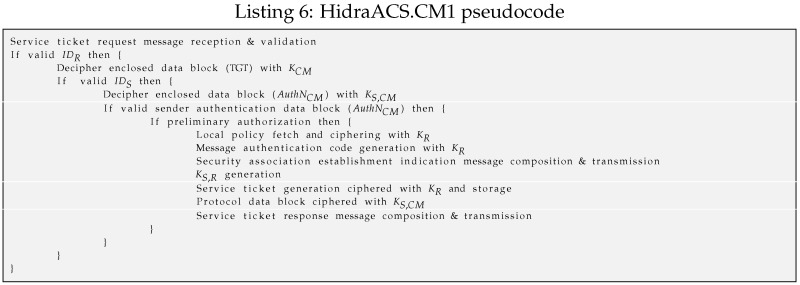
  – The CM2 module (*HidraACS.CM2*) is an auxiliary module of the CM1. It manages a key sequence for the assurance of the freshness of the policy provisioning message to avoid repetition attacks, as shown in Listing 7. For this purpose, a UDP socket is open at port 8866. It receives requests from the CDS when, after a parametrized set of trials of one-way functions over an initialized key, the CDS cannot properly validate the freshness of an indicative message HID_CM_IND from CM1. Therefore, once the validity of the request through the received MAC is checked, it sends back a new initial key value $K_{S,CM}^{i+1}$ and the MAC for validation in a responsive message HID_CM_IND_REP. 
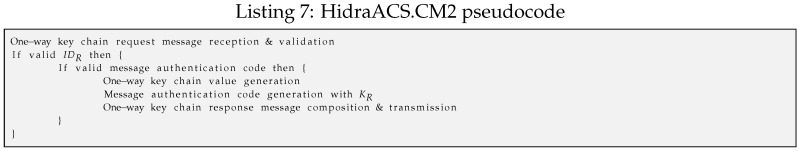
  – The LOG module (*HidraACS.LOG*) implements the reception of the log messages coming from the CDS, as shown in Listing 8. For this purpose, a UDP socket is open at port 8869. When it receives and checks the identity of the sending CDS of such a logging message, it updates the records and acknowledges back positively in a response message HID_R_ACK, thereby allowing the CDS to flush any related storage. 
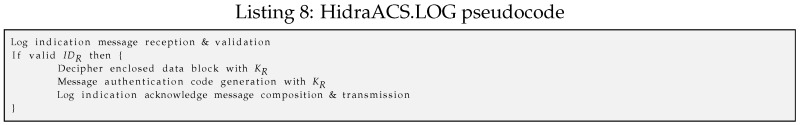
- Hidra in the subject side (*HidraS*) initiates the full sequence of the message exchange, as shown in Listing 9. It is implemented following a multi-step workflow. First, when no TGT has been obtained yet, it starts the authentication against the ANS in the ACS to obtain a TGT. It stores the nonces and key ($Nonce_{S,CM}$ and $K_{S,CM}$) required for the next service ticket request to the CM in the ACS.
  Second, when attempting to achieve security association establishment (SAE) with a CDS, HidraS sends a request of the service ticket to the CM using previous values to generate a message authenticator string.
  When it receives the response from the CM, it first parses and checks the validity of the addressed destination. Second, it deciphers the $K_{S,R}$ and some other nonces for freshness validation $Nonce_{2}$ and further CDS addressing $Nonce_{S,R}$. Third, it composes an access request for the CDS including the obtained service ticket and a message authenticator ciphered with $K_{S,R}$, and the module sends it.
  When it receives the response from the CDS, the module deciphers it and checks both the validity of the response with respect to the request and the session with respect to the service ticket. In the positive case, the security association is established, and the subject obtains a Subkey shared with the CDS, ready to be used in further access requests.
  From then on, and whenever it is locally authorized, any application on the subject side can access the resources on the CDS through the established secure session. 
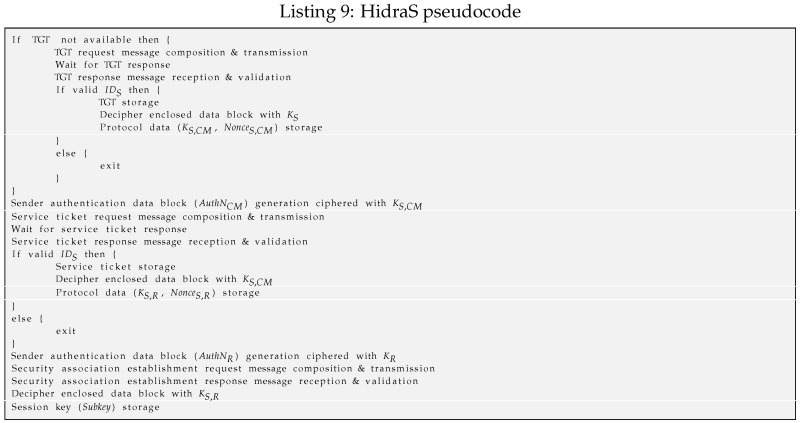
- Hidra on the CDS side (*HidraR*) is codified and installed as a server application, as shown in Listing 10. This server listens on a unique UDP port 8877 to be more efficient in terms of memory footprint. This server waits to receive four different messages, whose lengths are constant except for the indication message from the CM module in the ACS, which conveys an ad-hoc policy instance. 
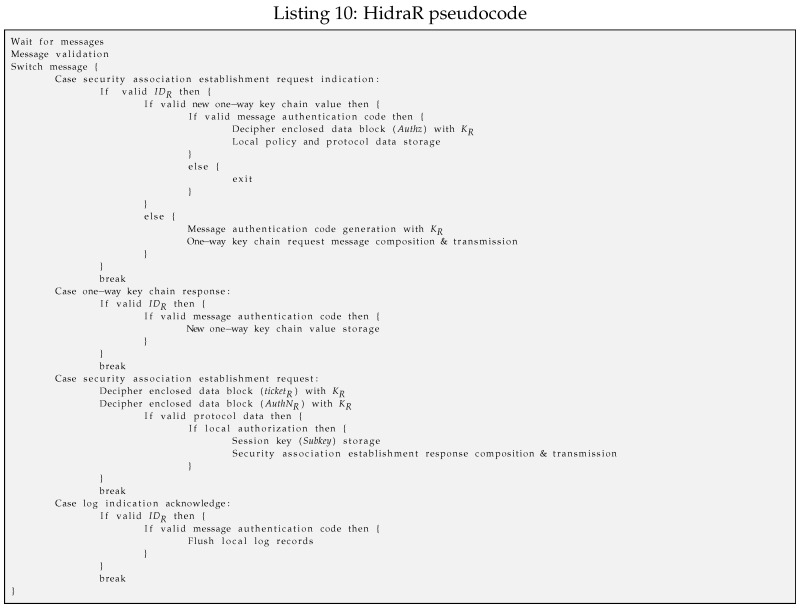
  – When an indication message HID_CM_IND is received from the ACS, it is first parsed, and both the addressed CDS as well as the MAC are validated. Then, the received key value $K_{S,CM}^{j}$ is validated and attempted for five iterations in unsuccessful cases. In the case of five unsuccessful trials using the received key value or when the chain is finished, a message HID_CM_IND_REQ is sent to the ACS to request a new sequence. In the positive case, the new key value as well as the subject identity and the shared $Nonce_{S,R}$ are stored for further message validation. Then, the module goes to the idle state.
  – When a response message HID_CM_IND_REP is received from the ACS initiating the key value chain, the message is validated after checking the identity of the CDS and $Nonce_{3}$; then, the received MAC is also confirmed. In the positive case, the new sequence key value is used to check the validity of the previous indication message from the ACS. Then, the module goes to the idle state.
  – When an access request message HID_S_R_REQ is received from the subject, the received ticket is first deciphered with $K_{R}$. The enclosed $K_{S,R}$ is used to decipher the message authenticator that conveys the $Nonce_{S,R}$ nonce to check the peers and the sequence of the messages. In the positive case, local authorization is enforced according to the related policy instance; in addition, the security association is established, and the shared Subkey is stored. Then, the response message to the subject is composed and sent. Finally, a new message for the ACS is composed, therein conveying the detailed log of the access attempt from the subject, which was previously ciphered with the $K_{R}$ key. Then, the module goes to the idle state.
  – When a log acknowledgment is received from the ACS, it is first parsed, the CDS id is checked, and the MAC is validated. In the positive case, log records can be flushed in the CDS, and the modules goes to the idle state.

During the development and codification, some design decisions have been made. Specifically,

- Every socket is UDP due to the high memory footprint of handling of TCP connections.
- In all the modules, upon reception of a message, once the message is identified, it is parsed, and the proper protocol-related checks, validations and reactions are performed following the specific Hidra protocol sequence.
- The modules acting as servers run in a continuous loop so that when any process on a message is finished or a message is discarded, they return to the initial state, subsequently waiting for the reception of new messages.
- When a message is discarded, the entire security association sequence is broken, and a subject would have to start from the resource ticket request point given that the TGT has not expired.
- The message cyphering is performed with AES-128, the block size is 16 bytes, and it is combined with a Ciphertext Stealing algorithm to avoid size increments with respect to clear-text messages.
- The cryptographic libraries are TomCrypt and tiny-AES-128 [39]. The latter library does not support the RC5 algorithm for the ciphering of the $AuthN_{CM}$, but its memory footprint is much lower than the former, and it is more appropriate for CDSs.

## 5. Performance Evaluation

In this section, the experimental performance analysis of an access control model for E2E security in CDSs is performed to demonstrate the establishment of a security association between a requesting subject and a CDS. The performance analysis covers the measuring and evaluation of the crucial performance parameters of such resource-constrained sensing environments.

Hereinafter, the crucial performance parameters are identified, and their measurement methods and computation are described. Finally, an analysis of the measurements is presented, therein describing and discussing the evaluation results.

The overall goal is to demonstrate the suitability of the designed access control model for CDSs in the envisioned scenarios.

### 5.1. Performance Metric Modeling

The experimental performance metric model used to conduct the evaluation of the impact of the reviewed access control model focuses on three critical parameters: (1) the response time of the access control model to establish an authorized E2E secure session; (2) the energy cost of this model for the protected CDS running on finite battery resources; and (3) the model’s impact on the local storage on the CDS and memory footprint.

Summarizing the acceptance criteria, the response time is required to be below accepted standard values if the proposal is to be useful, and the energy consumption, local storage, and memory footprint, due to the nature of the CDSs and their resource constraints, cannot exceed rational and proportional limits in the CDS if the proposal is to be feasible and scalable.

#### 5.1.1. Response Time Definition and Measuring Method

During the establishment of a security association, once a long-term TGT has been previously obtained by the subject, five messages are exchanged, as detailed in Section 3.3. Therefore, this response time includes the steps concerning when the subject requests and obtains the service ticket, the notification that the ticket is granted, the policy provisioning in the CDS by the ACS, and the security association request and response between the subject and the CDS.

To measure this time, a few code lines are inserted into the subject-side software code, therein applying two timestamps: one at the beginning of the security association establishment and the other at the end of this establishment.

#### 5.1.2. Energy Consumption Definition and Measuring Method

Regarding the energy cost measurement, the analysis focuses on the energy consumption of the CDS due to the exchange of five mandatory messages as well as two additional and occasional messages, which are the one-way key chain initiator message pair. Therefore, the energy consumed by the transmission and reception of bits over the air as well as the message processing consumption is considered.

For the measurement of the processing energy consumption, two timestamps are inserted at the sensor’s side: timestamps at the beginning and end of the message processing software code. Once the time to process each message is measured, a constant instantaneous power consumption ($P_{C}$) provided by the manufacturer in the data-sheet is considered to compute the energy consumption of each message.

For the computation of the power consumption due to the transmission and reception of each message, the involved message lengths in bytes and packet fragmentation are computed (considering 50 bytes as the longest IEEE 802.15.4 plus UDP/6LowPAN headers). The lengths of the messages exchanged during the authentication and authorization protocol range from 15 to 63 bytes. Enclosing the policy in the HID_CM_IND message (33 bytes), which is one of the smallest messages, implies the minimum fragmentation of 6LowPAN IPv6 packets over IEEE 802.15.4 links.

Additionally, the constant reception and transmission power consumption rates provided by the manufacturer in the data-sheet and a constant propagation bit rate are also considered. Table 7 shows the test-lab real-world (non-optimal) network data bit rate and the different instantaneous power consumption values used for the analysis. Note that these power consumption values correspond to a MEMSIC IRIS mote (XM2110CA) powered with a 3V power supply [37].

Finally, the power consumption is calculated as the sum of the individual power consumptions of each of the involved messages in the sensor.

#### 5.1.3. Storage and Memory Footprint

In this subsection, the increases in the permanent storage and memory footprint generated by the proposed access control model are considered. Specifically, the storage and memory footprints of the HidraR module, which handles up to seven messages, as described in Section 4.1.2, are considered. Such messages imply the exchange and processing of data blocks, such as identifiers, nonces, lifetimes, symmetric keys, tickets, authenticator blocks, MACs, and the most variable block, which is the provisioned policy instance.

On the one hand, regarding the permanent storage, which is usable after any reboot, the considered additional minimum entities, along with some original resources and sensing applications, are the following: the symmetric key shared with the ACS; the code used to run HidraR; the service ticket, including the records of active sessions; the last one-way key chain value; the access control policy; the session key shared with the subject; the session key, if any; and the logs pending the acknowledgment.

On the other hand, regarding the memory footprint, in addition to the permanently stored entities, some additional instantaneous data are required for the validations and freshness guaranties during the message exchanges. Some of these values are loaded and erased during the reception, processing and transmission of consecutive messages.

The measurement of the code, data storage and memory footprint is performed using a particular command, *size*, which provides both static and dynamic occupied memory amounts.

In this experimental implementation, the size command is executed in two cases, i.e., when HidraR is implemented and when connectivity has just been implemented, and the difference is measured.

### 5.2. Performance Analysis

In this section, the mean response time, the energy consumption and the local storage and memory footprint resulting from the trials are presented to establish a security association based on the subject under the implemented test-bed.

Concretely, series of 80 iterations have been defined to minimize the measurement as well as computation error. Moreover, due to the elasticity of the policy instances discussed in Section 3.2.1, the length of the policy provisioning message increases proportionally. Given this, several series have been launched to achieve a more accurate assessment of the impact of the length of the policy instances on the overall performance. This is done so that the results will support, on the one hand, the analysis of the impact of the tightness on the enforcement through the expressiveness of the policy and, on the other hand, a discussion on the effectiveness of the proposed policy codification method versus a generalized method such as CBOR.

First, the policy instances and lengths shown in Table 4 have been used as input in the trials. However, not all of them were successfully completed in the current test-bed. Thus, Table 8 shows a summary of the positively finalized trials in bold, whereas the infeasible trials are shadowed and in italics. The lengths of the JSON and JSON’ instances that are more clearly shadowed are feasible, but they have not been included in the presented tests and discussions for clarity purposes since they do not provide specific value or significantly differential measurements.

Therefore, since the lengths of the seven instantiated policies are (2, 7, 9, 14, 32, 81 and 123) bytes, the total lengths of the corresponding HID_CM_IND messages are (35, 40, 42, 47, 65, 103 and 156) bytes, respectively. These message lengths are considered in the energy consumption computation, as explained in the metric modeling above.

Second, Table 9 summarizes the schema of each of the series of 80 iterations performed to gather and process the measurements. Basically, prior to launch and the security association establishment by the subject, the correct policy instance needs to be configured in the ACS, and all the models need to be up and running. In the case of the successful finalization of the 80 iterations in a series, the resulting log files containing the raw measurements are processed to compute the derived measurements and to draw the graphical charts.

Additionally, the response time has been measured with two different configurations and one of the policy instances $IS_{1}$: one hop and two hops. Thus, the impact of the intermediate node insertion can be measured.

#### 5.2.1. Response Time

**Figure 7:**
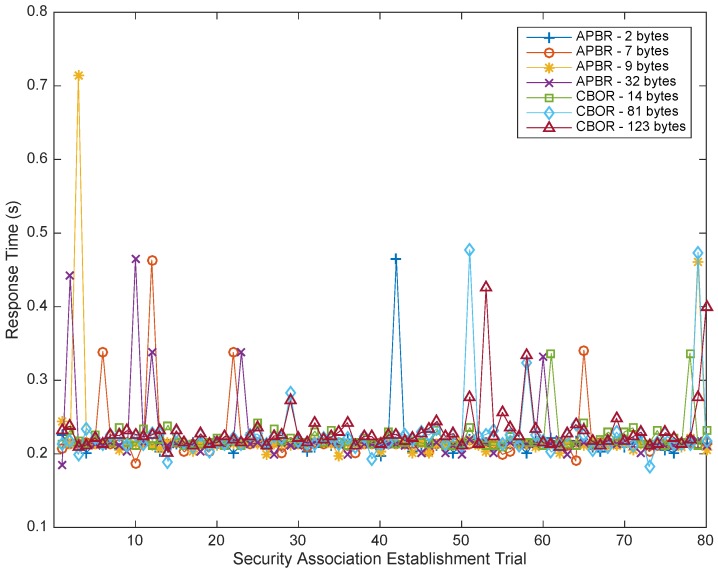
Response time of security association establishment considering up to seven series of 80 requests.

The resulting response time measurements after the launch of the seven feasible series with one and two hops are shown in Table 10. Such mean response times increase slightly with the length of the policy instances, as expected, ranging from 216 ms ($IS_{1}$ and APBR) to 232 ms ($IS_{3}$ and CBOR). To compare, Figure 7 shows the average response time with one hop, and regardless of sporadic picks, most response times remain in a constant, low range. This value is very good considering that the maximum acceptable delay in interactive E2E data transactions specified by Stallings [40] is 1000 ms.

In all cases, Figure 7 also indicates that the maximum response time in the worst sporadic case is below 720 ms and under the accepted quality standards for interactive applications.

**Figure 8:**
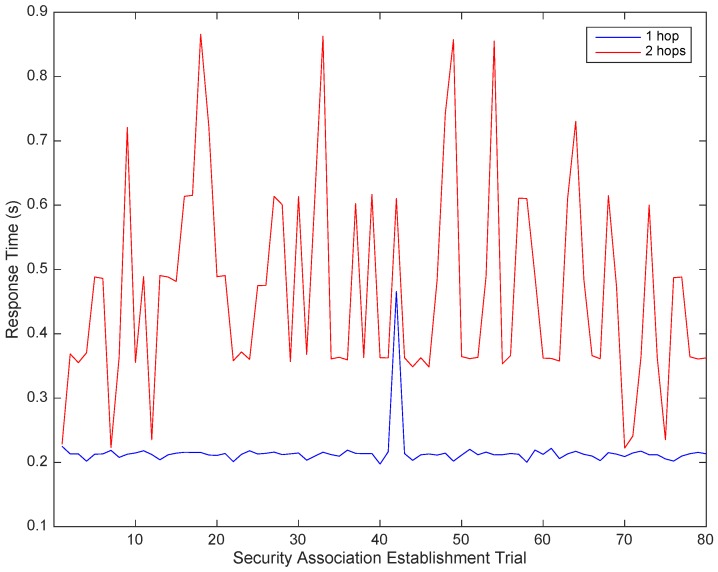
Response time of security association establishment considering two series of 80 requests with $IS_{1}$ in two network configurations: one and two hops.

The impact of Hidra depending on the network topology is analyzed under two network configurations: one and two hops. Figure 8 shows a composition of the measurements with the two configurations when a security association is established and the policy instance is $IS_{1}$. The accurately measured values of Table 10 indicate that a second hop results in a proportional increase of 240 ms on average. This value could also be considered as a referential increment per hop for further estimations aiming at large-scale deployments.

Beyond the absolute values, attempting to compare with similar existing measurements, one related comparable response time value has been found in the literature [41]. The measurements are performed by integrating C2 CDSs, and the analysis reveals that the response time for the authorization response starting when the subject sends the request is 480.96 ms (with one hop). The response time of Hidra is lower even with a worse network bit rate and, therefore, is better. In addition, in these comparison tests, there is no mention of additional performance indicators such as energy consumption.

#### 5.2.2. Energy Consumption

Considering the impact of the Hidra protocol on the energy consumption in the CDS, Table 11 shows the measured values of the power consumption related to seven series of 80 security association establishments. Such mean energy consumptions grow slightly with the length of the policy instances, as expected, ranging from 0.367 μAh ($IS_{1}$ and APBR) to 0.520 μAh ($IS_{3}$ and CBOR), which are very low values.

**Figure 9:**
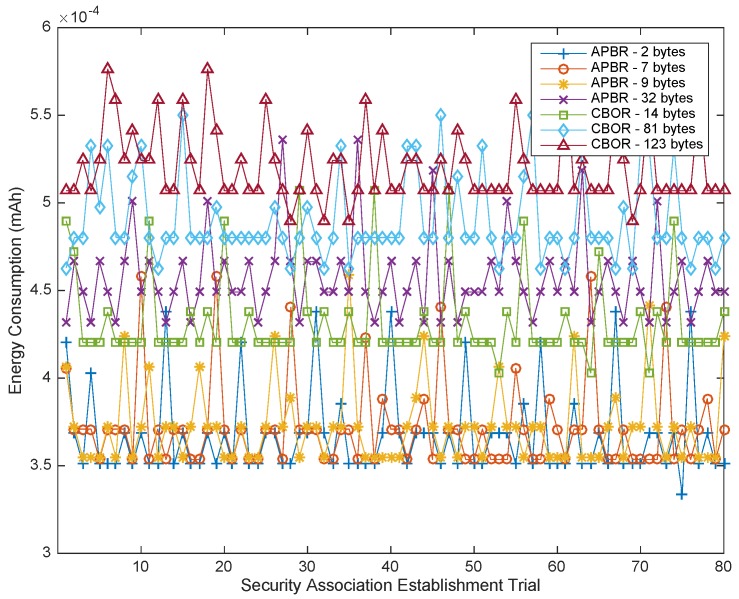
Energy consumption in security association establishment considering 80 requests in a one-hop network configuration.

**Figure 10:**
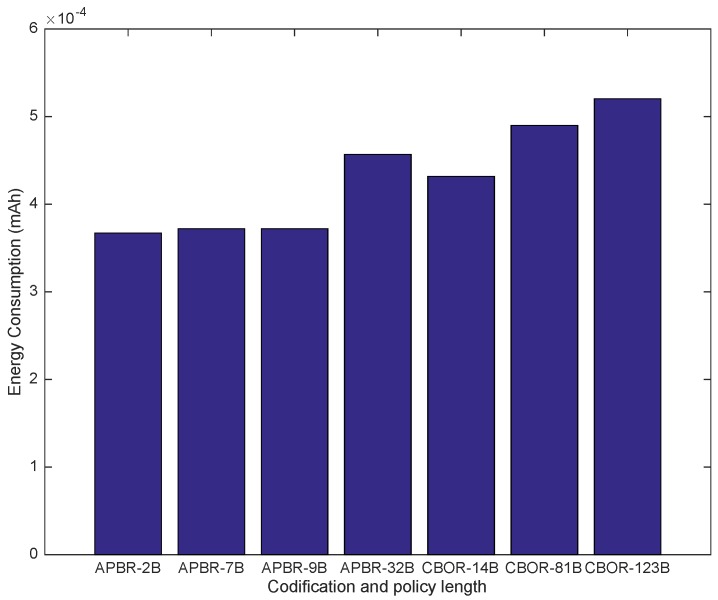
Energy consumption in security association establishment considering 80 requests in a one-hop network configuration.

Figure 9 shows that the measured average values of the power consumption remain below a very low maximum and are proportional to the length of the enforced policy instances. The proportionality is better appreciated in Figure 10, where a rising trend is depicted. These very good results mean that the impact of Hidra in terms of energy consumption is very low and that the high compression factor of the proposed policy codification is a critical success factor. Concretely, in the case of two AAA batteries with a capacity of 900 mAh, totaling 1800 mAh, considering a 95% battery performance, representing a real capacity of 1710 mAh, more than 4 million (4,291,006) requests could be handled during the battery lifetime.

In the envisioned scenario, the CDS is accessed by the subject to perform tasks such as personalization, parametrization, updating, upgrading, and maintenance. These types of interactions do not occur often. As the most exigent scenario, we can consider the case of different subjects requesting access every hour to tune the user experience in an application whereby the users change hourly.

In this most exigent scenario, a subject making one request per hour, totaling 24 requests per day, 8760 requests per year, could obtain a response for approximately 490 years (489.840) with the given battery life. Therefore, the energy consumption of Hidra could be ignored, and the battery life would depend basically on the main application purpose of the CDS.

#### 5.2.3. Storage and Memory Footprint

Finally, from the storage point of view, at the CDS, the amount of RAM is the most limiting aspect, compared with permanent storage, which is usually an order of magnitude larger. Table 12 shows the comparative memory footprint between the full HidraR software and just the connectivity stack, where text is the program size, data is the initialized RAM size, and bss is the uninitialized pre-zeroed RAM (e.g., buffers).

Assuming that the sizes are dependent on the programming style, measurements of the increment on the storage occupancy are 18,544 bytes, and the memory footprint is 1450 bytes.

Therefore, although dependent on the specific implementation, the impact is considered acceptable considering the available 128 kB and 8 kB of flash and RAM memory in such constrained devices, i.e., C0 CDSs, such as those used in the implementation [37]. One additional remarkable result is the considerable influence of the activated debugging and measuring instructions on the final footprint measurements.

## 6. Conclusions

Smart objects enabling the envisioned pervasive scenarios and advanced use cases are expected to provide services toward achieving improved user experience, higher interoperability and greater manageability. In such environments, where massive deployments are expected, smart things are inexpensive and thus implemented in devices with constrained resources. However, they are also critical components since they handle essential information that needs to be available, keeping the confidentiality and integrity; therefore, security needs to be ensured in an efficient manner. Current approaches addressing the principle of least privilege, based on the expressiveness and updating of the policy to be enforced in the sensors, are challenged by feasibility constraints.

The conducted performance evaluation is focused on an innovative access control model based on a hybrid architecture and a policy language for dynamic fine-grained policy enforcement in the sensor. Such policy enforcement is based on local context conditions and corresponding obligations not only during secure session establishment but also afterward, when the security association is in use, to control the behavior of the access. Such a dynamic policy cycle avoiding local storage is enabled by an efficient message exchange protocol named Hidra. The Hidra protocol assures mutual authentication, expressive policy injection, tight policy enforcement in the secure association establishment and derived resource access, as well as the accounting for further tracking and auditing purposes.

The proposed feasibility assessment is based on a test-bed implementation of an innovative access control model for very-constrained devices: C0 and C1 CDSs. The experimental performance analysis, focusing on three key performance indicators, i.e., the response time, the power consumption and the memory footprint, provides remarkable results. Based on these measurements, the performance evaluation of this proposal demonstrates the feasibility of this analyzed access control model for resource-constrained sensors.

## Figures and Tables

**Table 1:** EBNF representation of a schematic policy.

*policy ::= ’{’ ’"id"’ ’:’ integer ’,’ ’"effect"’ ’:’ ( DENY | PERMIT ) ( ’,’ ’"ruleset"’ ’:’ ’[’ rule+ ’]’ )? ’}’*

**Table 2:** Policy instance sample groups and currently implemented platforms, excluding the proposed access control model.

Sample	Key Features	Implemented Platforms	Rules	Conditions	Inputs	Obligations	Re-Evaluation
S1	No local rules	C0-C2	none	none	none	none	no
S2	Local conditions	C1-C2	one	many	many	none	no
S3	Local conditions	C2	one	many	many	many	no
	and obligations						
S4	Several rules	C2	several	many	many	many	yes

**Table 3:** Examples of policy instance samples.

Sample	Rules	Conditions	Inputs	Obligations	Re-Evaluation	Length (Bits)	Length (Bytes)
$IS_{1}$	0	0	0	0	no	10	2
$IS_{2}$	1	1	1	0	no	53	7
$IS_{3}$	1	1	1	1	no	67	9
$IS_{4}$	2	3 + 3	1 + 1	1 + 1	yes	258	32

**Table 4:** Length comparison for different representations of four examples of sample instances.

Representation	Nature	Length (Bytes)			
		${\mathrm{IS}}_{1}$	${\mathrm{IS}}_{2}$	${\mathrm{IS}}_{3}$	${\mathrm{IS}}_{4}$
JSON	Human-readable text	30	164	236	798
JSON’	Pre-processed text	23	118	174	554
CBOR	Binary stream	14	81	123	391
APBR	Optimized binary stream	2	7	9	32

**Table 5:** Comparison summary of operating systems for Hidra test-bed implementation.

OS	Kernel	Network Stack	Programming Model	Development Language	Scheduling	Real-Time	Adoption & Support
TinyOS	Monolithic	Yes	Event-driven	NesC	Preemptive	No	High
Contiki OS	Modular	Yes	Protothreats	subset of C	Preemptive	Partial	High plus
Riot	MicroKernel	Yes	Multi-threating	C, C++	Priority-based	Yes	High

**Table 6:** Hardware technical features.

	TelosB	Iris
**Processor**	TI MSP430, 16 bit	XM2110CA, 8 bit
**Program Flash**	48 kB	128 kB
**Measurement Serial Flash**	1024 kB	512 kB
**RAM**	10 kB	8 kB
**Serial communications**	UART / USB	UART / MIB520CB
**RF transceiver**	CC2420	CC2520
**RF frequency band**	2400 to 2483.5 MHz	2405 to 2480 MHz
**Current draw / Rx**	23 mA	16 mA
**Transmit data rate**	250 kbps	250 kbps
**Outdoor range**	75 to 100 m	300 m
**Indoor range**	20 to 30 m	50 m
**Battery**	2X AA batteries	2X AA batteries
**User interface**	3 LEDs	3 LEDs
**Sensors**	Light, humidity, temperature	Light, humidity, temperature

**Table 7:** Parameters used to characterize the energy consumption of sensor nodes.

Name	Description	Value
$B_{N}$	Effective network wireless link data bit rate	70 kbps
$P_{RX}$	Power consumption in reception mode	48 mW
$P_{TX}$	Power consumption in transmission mode (3 dBm)	51 mW
$P_{C}$	Power consumption in message processing mode	8 mW

**Table 8:** Length summaries of four examples of sample instances, as well as their feasibility in the current test-bed implementation.

Representation	Nature	Length (Bytes)			
		${\mathrm{IS}}_{1}$	${\mathrm{IS}}_{2}$	${\mathrm{IS}}_{3}$	${\mathrm{IS}}_{4}$
JSON	Human-readable text	30	*164*	*236*	*798*
JSON’	Pre-processed text	23	118	*174*	*554*
CBOR	Binary stream	**14**	**81**	**123**	*391*
APBR	Optimized binary stream	**2**	**7**	**9**	**32**

**Table 9:** Security association establishment trial series launching file.

**Requirements**	1.- Measuring and debugging commands need to be active in the three Hidra modules: HidraACS, HidraS and HidraR. 2.- HidraACS module must be configured to choose proper IS policy instance for each of the series of 80 iterations. 3.- HidraACS on the ACS and HidraR on the CDS need to be started and wait for messages on corresponding UDP ports. 4.- The remaining border router and intermediate sensor nodes need to be active to assure E2E IPv6 network connectivity.
**Procedure**	1.- Launch HidraS module. 2.- In the case of successful series bulk, log files to a unique wrapping file for further computations.
**Inputs**	For each of the series, one IS policy instance conveyed in Table 8 is used.
**Pass/Fail criteria**	When the full series of 80 iterative security association establishment finishes correctly, the trial is considered successful.
**Expected output**	Each of the Hidra modules generates a log file with the timestamps, ticks and raw measurements for further treatment. 1.- Log file with 80 measurements of response times registered by HidraS. 2.- Log file with sets of ticks from starting to ending instants of message processing registered by HidraR. The output individual log files are wrapped into a unique log file to be processed and represented by a graphical generation tool.
**Observations**	The wrapping format is Excel, and the graphics are generated with Matlab.

**Table 10:** Response time measures.

IS	Format	Network (Hops)	Length (Byte)	Average (ms)	Median (ms)	Standard Deviation (ms)	95% Configuration Int. (ms)
$IS_{1}$	APBR	1	2	215.39	213.09	28.79	(209.08,221.70)
$IS_{1}$	APBR	2	2	357.56	343.97	183.44	(317.37,397.76)
$IS_{2}$	APBR	1	7	219.92	213.60	37.15	(211.78,228.06)
$IS_{3}$	APBR	1	9	221.80	213.55	62.54	(208.10,235.51)
$IS_{4}$	APBR	1	32	222.48	213.37	44.51	(212.73,232.23)
$IS_{1}$	CBOR	1	14	221.98	215.28	20.35	(217.51,226.44)
$IS_{2}$	CBOR	1	81	226.73	219.49	43.54	(217.19,236.28)
$IS_{3}$	CBOR	1	123	231.51	223.73	34.63	(223.92,239.10)

**Table 11:** Energy consumption measures.

IS	Format	Length (Byte)	Average (μAh)	Median (μAh)	Standard Deviation (μAh)	95% Configuration Int. (μAh)
$IS_{1}$	APBR	2	0.3671	0.3510	0.0491	(0.3563,0.3779)
$IS_{2}$	APBR	7	0.3720	0.3709	0.0246	(0.3666,0.3774)
$IS_{3}$	APBR	9	0.3722	0.3719	0.0123	(0.3694,0.3748)
$IS_{4}$	APBR	32	0.4567	0.4491	0.0122	(0.4541,0.4594)
$IS_{1}$	CBOR	14	0.4316	0.4204	0.0368	(0.4236,0.4397)
$IS_{2}$	CBOR	81	0.4898	0.4803	0.0122	(0.4871,0.4925)
$IS_{3}$	CBOR	123	0.5204	0.5069	0.0122	(0.5179,0.5233)

**Table 12:** Footprint increment through *size* command.

Version	Text	Data	Bss
HidraR	63,940	1548	5682
Only connectivity	45,396	338	4442
Difference	18,544	1210	1240

## Supplementary Materials

The following are available online at https://www.youtube.com/watch?v=sWt4pMLjRJ0&t=5s, Video S1: Expressive-Policy-based access control for resource-constrained sensors.

## Abbreviations

The following abbreviations are used in this manuscript:

ABAC	Attribute-based access control
ACS	Access Control server
ANS	Authentication server
APBR	Authorization Policy Binary Representation
AZS	Authorization server
CBOR	Concise binary object representation
CDS	Sensor or actuator in constrained device
CM	Credential manager
CoAP	Constrained application protocol
DCAF	Delegated CoAP authentication and authorization framework
DCApBAC	Distributed capability-based access control
EBNF	Extended Backus-Naur Form
E2E	End to end
IETF	Internet Engineering Task Force
IoT	Internet of things
IS	Instance Sample
JSON	Javascript object notation
MAC	Message authentication code
MCU	Micro-controller Unit
PDM	Policy domain model
PKC	Publick key cryptography
RBAC	Role-based access control
SKC	Simmetric key cryptography
TGT	Ticket granting ticket
XACML	Extensible access control markup language
XML	Extensible markup language
